# Tissue microarray analysis delineate potential prognostic role of Annexin A7 in prostate cancer progression

**DOI:** 10.1371/journal.pone.0205837

**Published:** 2018-10-15

**Authors:** Ximena Leighton, Alakesh Bera, Ofer Eidelman, Lukas Bubendorf, Tobias Zellweger, Jaideep Banerjee, Edward P. Gelmann, Harvey B. Pollard, Meera Srivastava

**Affiliations:** 1 Department of Anatomy, Physiology and Genetics, and Institute for Molecular Medicine, Uniformed Services University School of Medicine (USUHS), Bethesda, MD, United States of America; 2 Institute for Pathology, University Hospital Basel, Basel, Switzerland; 3 Division of Urology, St. Clara Hospital, Basel, Switzerland; 4 George Washington University, Washington, D.C., United States of America; 5 Department of Medicine, Colombia University Medical Center, New York, NY, United States of America; University of South Alabama Mitchell Cancer Institute, UNITED STATES

## Abstract

**Background:**

Annexin A7 (ANXA7) is a member of the multifunctional calcium or phospholipid-binding annexin gene family. While low levels of ANXA7 are associated with aggressive types of cancer, the clinical impact of ANXA7 in prostate cancer remains unclear. Tissue microarrays (TMA) have revealed several new molecular markers in human tumors. Herein, we have identified the prognostic impact of ANXA7 in a prostate cancer using a tissue microarray containing 637 different specimens.

**Methods:**

The patients were diagnosed with prostate cancer and long-term follow-up information on progression (median 5.3 years), tumor-specific and overall survival data (median 5.9 years) were available. Expression of Ki67, Bcl-2, p53, CD-10 (neutral endopeptidase), syndecan-1 (CD-138) and ANXA7 were analyzed by immunohistochemistry.

**Results:**

A bimodal distribution of ANXA7 was observed. Tumors expressing either high or no ANXA7 were found to be associated with poor prognosis. However, ANXA7 at an optimal level, in between high and no ANXA7 expression, had a better prognosis. This correlated with low Ki67, Bcl-2, p53 and high syndecan-1 which are known predictors of early recurrence. At Gleason grade 3, ANXA7 is an independent predictor of poor overall survival with a p-value of 0.003. Neoadjuvant hormonal therapy, which is known to be associated with overexpression of Bcl-2 and inhibition of Ki67 LI and CD-10, was found to be associated with under-expression of ANXA7.

**Conclusions:**

The results of this TMA study identified ANXA7 as a new prognostic factor and indicates a bimodal correlation to tumor progression.

## Introduction

The rising incidence of prostate cancer in the U.S. and the mortality associated with this cancer represents a significant health risk to Americans. Despite recent intensive research investigations, much remains to be learned about specific molecular defects associated with prostate cancer onset and progression and the clinical course of this tumor shows a considerable variability due to its biological heterogeneity [1–5]. The identification of new prognostic markers in prostate cancer is therefore essential to predict the individual outcome. Alas, no specific and well-characterized molecular marker has been uniformly recommended for routine application for the detection of metastasis and recurrence for prostate cancer [6–8].

Tissue microarray technology allows for rapid molecular profiling of large numbers of tumors in a single experiment [9]. The prognostic utility for TMAs has been demonstrated in various human cancers such as breast [10], urinary bladder [11], and kidney cancer [12]. Previously, we reported a larger prognostic TMA containing tissue samples of prostate cancers from 551 patients with long-term follow-up on progression, overall and tumor-specific survival and identified the prognostic significance of syndecan-1 in prostate cancer, as well as a downregulation of CD-10 after neoadjuvant hormonal therapy [13]. In this study, we identified Annexin A7 as a potential candidate biomarker and analyzed its expression levels with respect to its prognostic impact. In addition, we correlated the results with the expression of the molecular markers (apoptotic and tumor survival) Ki67, Bcl-2, CD10 and syndecan-1 (CD138) for which a prognostic significance in prostate cancer has previously been suggested [14–19]. All of these tumor markers are very significant for proliferation and apoptosis in predicting survival of different cancers including breast, prostate and colorectal cancers [20]. The human ANXA7 (genetic position at 10q21) has displayed a tumor suppressor role in multiple *in vivo* and *in vitro* studies involving prostate cancer samples [21–23]. For instance, the expression level of ANXA7 (mRNA or protein level) is quite high in normal prostate tissue [24]. Besides, our *ANXA7*^+/-^ mice study has demonstrated a cancer-prone phenotype, developing a spectrum of tumors including prostate cancer [25]. Furthermore, in our multi-tumor tissue microarray study, we found that ANXA7 protein expression was repetitively decreased in several different tumor tissues, demonstrating a typical tumor suppressor gene pattern, with a specific reduction in androgen-resistant prostate cancers [21, 23]. Altogether, these results point towards a significant prognostic impact of ANXA7 in prostate cancer and warrant further investigation.

## Materials and methods

### Ethics statement

This particular study was performed with the tumor samples which were collected between year 1971 to 1996, from two Kaiser Hospitals in Portland, OR, and is described in detail by Zellweger *et al*.[13]. Samples were collected through maintaining proper protocol. Use of the tumor material had been approved by the Institutional Review Board of the Kaiser Foundation Hospital, Center for health Research, Oregon, effective December 15, 2005. Since this was a retrospective study with fully anonymized patient data, there was no need for written informed consent. Besides, there were no subjects (patients) included lower than age of eighteen for this research.

### Prostate cancer patients and preparation of tissue specimens

The tissue microarray (TMA) from prostate cancer patients was constructed as previously described [9, 13]. A total of 551 patient samples (all members of the Kaiser Foundation Health Plan) were analyzed with a median age of 63.6 (range 45–92) years. The patients were treated for clinically localized prostate cancer by radical prostatectomy or transurethral resection (TURP) [13]. Besides, a complete follow-up data was also available for all patients, including progression specifications. The overall survival was 5.9 years (median), with a range 0.5–20 years; and the tumor-specific survival value was 5.9 years (median), range 0.5–20 years [13]. Progression was determined by increasing serum prostate-specific antigen (PSA) concentrations. The collected tumor specimens were segregated into two different sub-groups–radical prostatectomy (498 samples) and transurethral resections (TURP) (53 samples). As an experimental requirement, the slide with the least distinguished tumor area was selected for TMA experiment [13]. As described earlier, because of their small size, Gleason grade rather than Gleason score was considered for the TMA sections [13, 26]. For the further stage distribution, the radical prostatectomy specimens were assigned as pT2 (396 patients), pT3 (86 patients), and pT4 (16 patients) according to the criteria of the International Union against Cancer and the American Joint Commission on Cancer [27].

### Neoadjuvant hormonal treatment patients

We also collected and processed the prostate cancer tissues from neoadjuvant hormonal treatment patients’ specimen. As described earlier, the neoadjuvant hormonal treatment (typically Leuprolide given monthly for 1–3 doses) was given to 101 of 498 patients who eventually underwent radical prostatectomy process [13]. For control experiments, the nonmalignant 86 control tissues specimens were collected from patients with benign prostatic hyperplasia (BPH).

### Tissue-microarray immunohistochemistry

The immune-histochemical assays were performed as described earlier [13]. In brief, one of the six replicate blocks were used for immune-histochemical staining. Next, the standard indirect immune-peroxidase procedures were performed (ABC-Elite, Vector Laboratories) [13]. The monoclonal antibodies were used for immune-histochemical staining to detect ANXA7 (1:100, BD Biosciences, monoclonal (Clone 5), Cat# 610668)), Ki67 (MIB1,1:800; DAKO, Glostrup, Denmark), Bcl-2 (124-BCL-2, 1:400, DAKO, Glostrup, Denmark), p53 (DO-7, 1:200, DAKO, Glostrup, Denmark), CD-10 (56C6, 1:50, NOVOCASTRA), and syndecan-1 (CD-138; MI15, 1:200, DAKO, Glostrup, Denmark). The treatment performed for the antigen retrieval in a microwave (ANXA7, Bcl-2, p53, CD-10, and syndecan-1) or a pressure cooker (Ki67). Diaminobenzidine was used as a chromogen. The intensity of immune-staining for ANXA7, p53, Bcl-2, CD-10, and syndecan-1 was visually scored and segregated into four different groups (negative, weak, moderate, and strong signal). To designate over-expression of a certain protein, we considered at least moderate IHC intensity should observe over 10% of tumor cells [13]. As described earlier, for the protein level expression of Ki67 and p53, only nuclear staining was considered for the analysis [13].

### Statistical analysis

The statistical analysis was performed as described previously [13]. The correlations between expression of different markers (ANXA7, Bcl-2, p53, etc) and Gleason grades were determined by Chi-square tests. Kaplan-Meier analyses were performed to plot the survival curves. Patients were censored based on the time of their last clinical data or the date of nontumor-related death. The data analysis and statistical values were validated based on survival analysis as described by Bewick et al. [28]. The levels of statistical significance were considered based on the p-value (log rank) at least at *p* < 0.05, and all statistical calculations were performed using SAS JMP 3.0 software [13].

## Results

### Prognostic impact of ANXA7 expression

**Fig 1:**
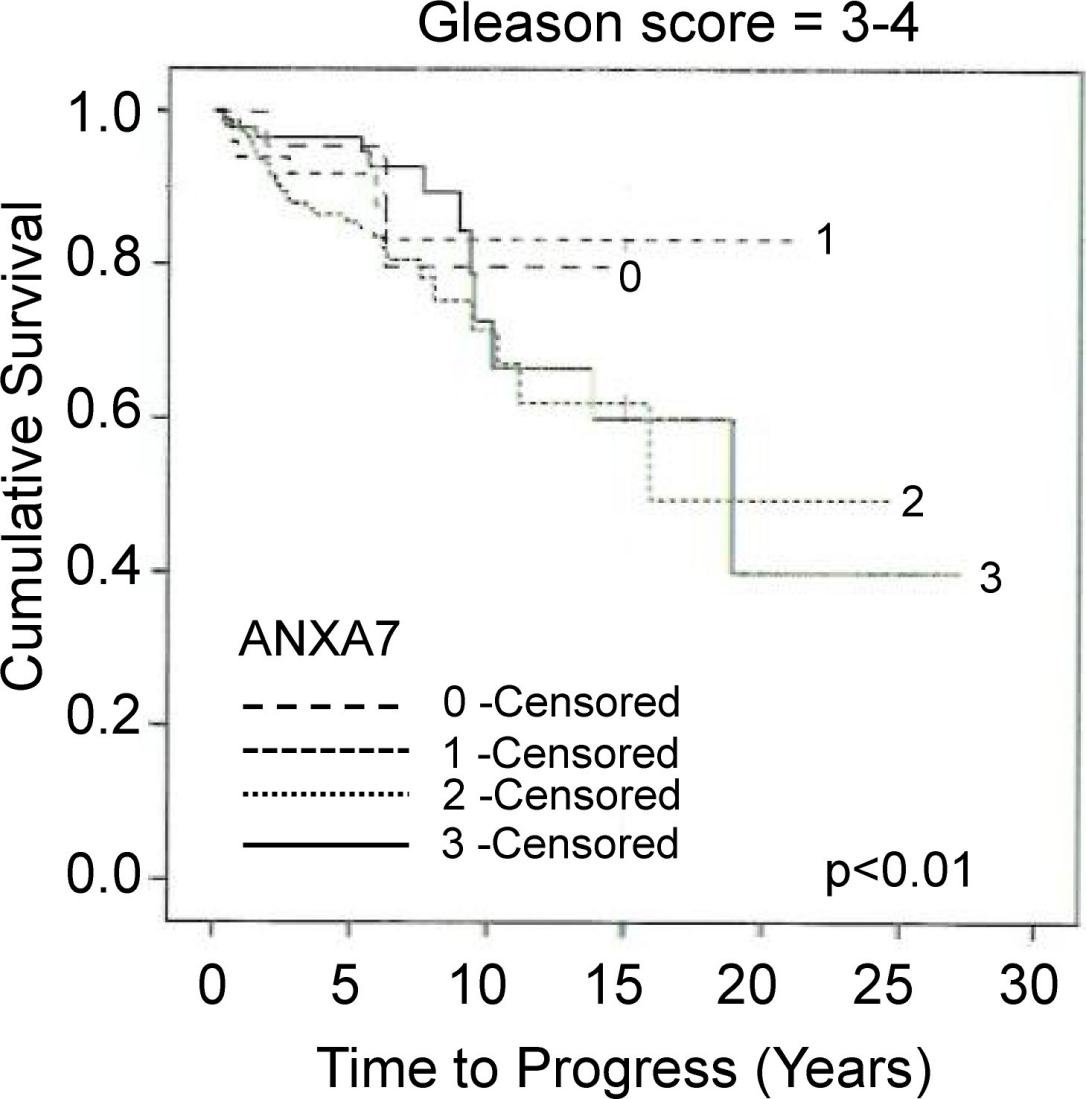
The Gleason grades were determined by independently asses on tissue microarray (TMA) and indicating the cumulative survival. The lower Gleason grades were not significantly correlated with the ANXA7 expression; however, we found a significant correlation at the Gleason scores of 3 and 4. The total number of tumors for the Gleason score (3–4) is fourteen (n = 14).

**Fig 2:**
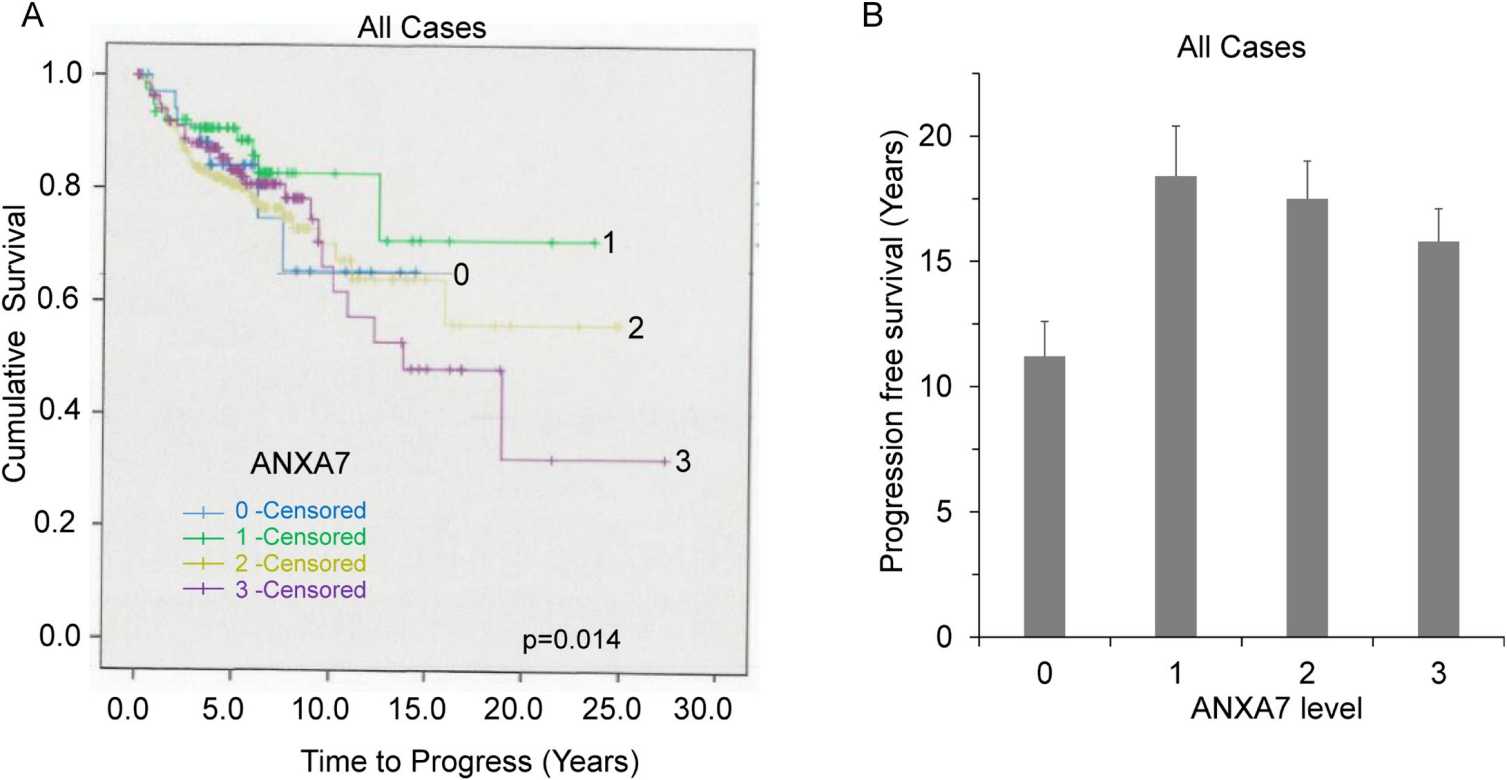
The Kaplan-Meier survival curve compares 551 prostate cancer patient samples along with 86 benign prostate tissue. **A.** The differential expression level of ANXA7 is associated with cumulative survival. Threw independent assays, the Gleason grades were determined on tissue microarray and indicating the time to progression, overall and tumor-specific survival. **B**. The graph represents the survival rate with different expression levels of ANXA7. The no (overall n = 436) of total case and differential expression for each ANXA7 score are described in the Table 1.

A total of 551 patients with prostate cancer and long-term follow-up information were evaluated using immunohistochemistry on a prognostic Tissue Microarray. The disease progression (median 5.3 years), tumor-specific and overall survival (median 5.9 years) were included in this study. For the controls, there were eighty-six specimens from benign prostatic hyperplasia included. Kaplan-Meier survival curves were constructed to compare the patients with positive ANXA7 staining to those with negative ANXA7 staining. First, we tried to segregate the tumor grades based on ANXA7 expression level. The ANXA7 expression levels did not show significant differences in the lower Gleason grades. There were a total of fourteen (n = 14) cases (tumor samples) that showed these Gleason scores (score = 3–4). We observed the correlation of ANXA7 expression levels at Gleason score 3 and 4 (**Fig 1**). We found an approximate fifteen years of survival rate ~ 78% and ~83% at the ANXA7 expression levels 0 and one, respectively. Whereas, at ANXA7 expression levels 2 and 3, the fifteen years survival rate show decrease levels, ~ 58% and 60%, respectively (**Fig 1**) with a very significant low p-value (ANXA7 (0–3)(1–2) = p<0.01). The presence of ANXA7 protein level in each of these patient specimens was correlated with patient survival parameters. Four types of ANXA7 expression can be discriminated in the prostate cancer specimens. These groups are designated “0” for negative or very low ANXA7 expression; “1” for weak ANXA7 expression; “2” for moderate ANXA7 expression; and “3” for strong ANXA7 expression. As shown in **Fig 2A and 2B**, Kaplan-Meier curves of univariate cumulative survival in patients with low (0) versus high (3) cytoplasmic ANXA7 expression show a significant separation within 15 years of follow-up. The fifteen-year survival is 65% for group 0 (negative or very low ANXA7 expression, n = 35) and group 2 (higher ANXA 7 expression, n = 205), and 45% for group 3 (highest ANXA7 expression, n = 125). However, for group 1 (moderate or optimal ANXA7 expression) survival is up to 70% (p-value = 0.014, log-rank test). The overall number of cases (n) and the individual ANXA7 expression cases (scores 0–3) were presented at Table 1. Therefore, we observed a bimodal distribution of ANXA7, tumors expressing both high (3) or no (0) ANXA7 were found to be associated with poor prognosis. However, ANXA7 at an optimal level (1) is associated with good prognosis.

**Table 1:** The distribution of tumor numbers or case processing summery was presented for different ANXA7 expression sores. Total number of tumors designated as overall cases.

Case processing Summary	Expression Score ANXA7	Numbers (n)
	0	35
*All cases*	1	71
(ANXA7 at	2	205
various Gleason scores)	3	125
	**Overall**	**436**
*ANXA7 and Syndecan-1*		
	0	10
Syndecan -1	1	44
Neg	2	131
	3	89
	**Overall**	**274**
	0	21
Syndecan -1	1	25
Positive	2	69
	3	35
	**Overall**	**150**
*Neoadjuvant*		
	0	32
	1	51
Treatment: No	2	171
	3	104
	**Overall**	**358**
	0	3
Treatment: Yes	1	20
	2	33
	3	21
	**Overall**	**77**

### Analysis of ANXA7 and its correlation with other clinicopathological factors such as Ki67, Bcl-2, CD-10 and syndecan-1

**Fig 3:**
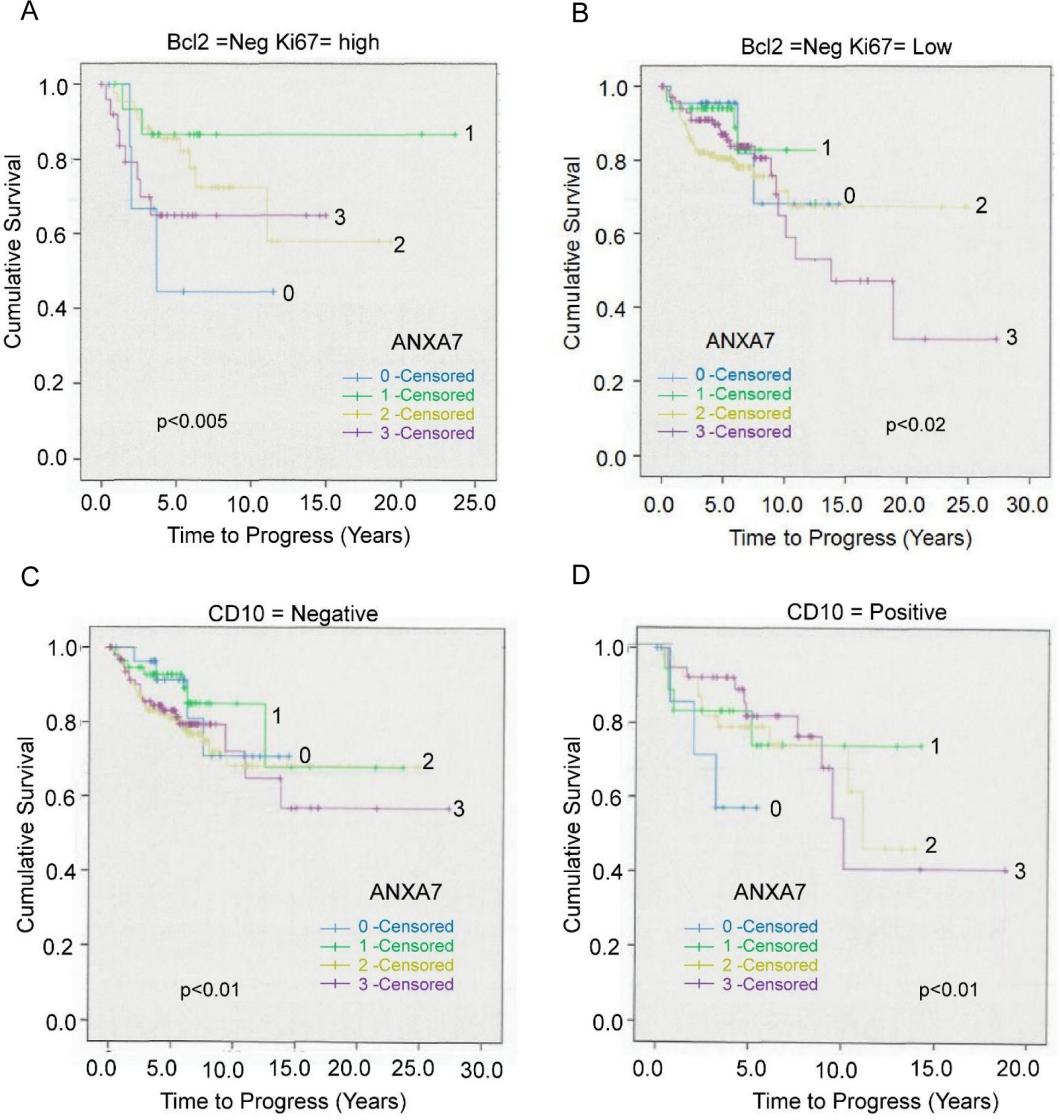
The correlation between survival markers such as Bcl-2, Ki67, CD10 and the ANXA7 expression levels. The Kaplan-Meier curve represented the time of cumulative survival with respect to different proliferation markers. **A.** Representation of the cumulative survival associated with Bcl-2 negative and Ki67 high tissues with different levels of ANXA7 expressions (total cases, n = 56). **B.** A similar cumulative survival graph was represented with an association of Bcl-2 negative but Ki67 low tissues with different ANXA7 expression levels (total cases, n = 363). **C-D.** The expression of ANXA7 and survival of prostate cancer patients with respect to CD-10 expression. The expression of CD-10 is linked to a decrease in the overall survival rate. Herein, we demonstrated how the ANXA7 expression levels related to differential survival rates and expression levels of CD-10, negative (panel C) or positive (panel D).

We analyzed the expression level of ANXA7 in prostate cancer tissue arrays and compared with the co-expression of other tumor apoptotic or proliferation regulating factors including Bcl-2, CD-10, p53 and syndecan-1. The cytoplasmic Bcl-2 over-expression was found in prostate cancer specimens and predicted early tumor progression [13, 29–31]. In this current study, we investigated a combination of Ki67 and ANXA7 expression at low Bcl-2 expression. In tissues where Ki67 was high, the highest survival rate was observed at optimal ANXA7 expression (level 1, n = 7, ~90% 10-year survival, Overall cases, n = 56), as compared to ~40–60% for either high (level 3, n = 12) or no (level 0, n = 7) ANXA7 expression. The log rank p-value between different ANXA7 scores (ANXA7 (0–3)(1–2)) was very significant (p = 0.005). In tissues where Ki67 was low, the highest survival rate was observed at optimal ANXA7 expression (level 1, n = 62, ~85% 10 year survival; Overall case, n = 363), as compared to ~70% for no or ~ 50% for high (level 3, n = 108) ANXA7 expression (**Fig 3A and 3B**). Thus, the combined analysis of Bcl-2 expression and Ki67 revealed that the survival was not favorable in Bcl-2 negative tumors with low Ki67 and high ANXA7 (**Fig 3B**), but increased with the moderate expression of ANXA7. However, the poor survival was associated with low ANXA7 expression (p<0.005, **Fig 3A**) and low Bcl-2 along with high Ki67 (p<0.02) expression levels. Thus, the ANXA7 expression level showed a bimodal representation toward cumulative survival in terms of Bcl2 and Ki67 expression.

We also analyzed the expression of ANXA7 and survival of prostate cancer patients with respect to CD-10 expression. The expression of CD-10 is linked to a decrease in the overall survival rate [14]. When CD10 expression is positive, ANXA7 expression at level 1 can significantly improve cumulative survival. However, a high ANXA7 (level 3) showed a 10 year survival rate of ~40% while a negative ANXA7 showed a survival rate of ~55% (level 2), while an optimal level of ANXA7 (level 1) showed a survival rate of ~70% (p<0.01; **Fig 3C and 3D**)–pointing again to a bimodal distribution of the effects of ANXA7 expression on survival rate.

### Cross-talk between p53, syndecan-1 and ANXA7

**Fig 4:**
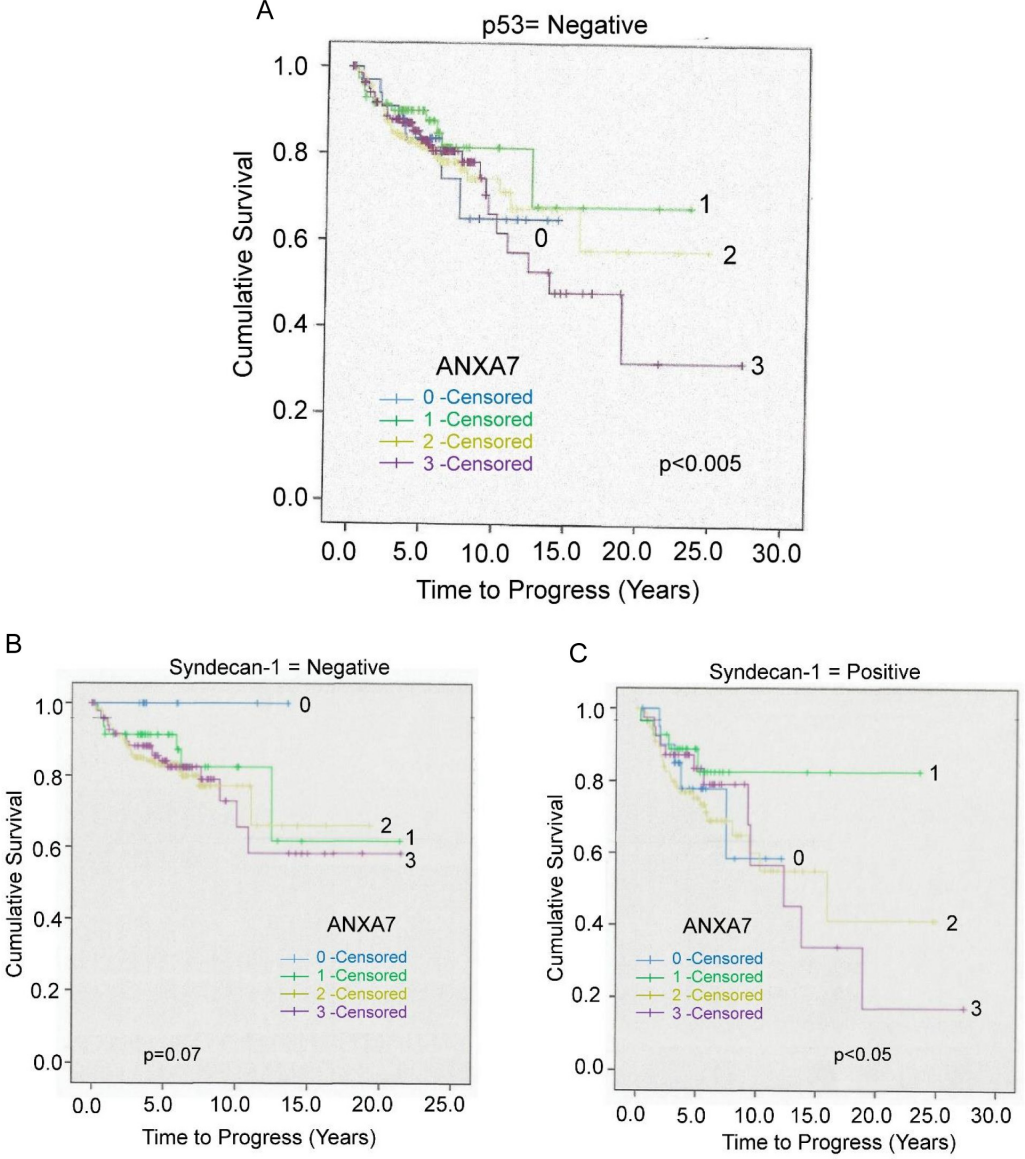
The Kaplan-Meier curve correlated the time to tumor-specific survival with respect to ANXA7 expression levels and prostate tumor-specific markers such as p53 and syndecan-1. **A.** The graphical representation of cumulative survival was linked to low p53 and different levels of ANXA7 expression (total cases, n = 417). **B.** The description of a low expression level of syndecan-1 is associated with cumulative survival with the differential expression of ANXA7. **C**. The deferential survival curve was associated with positive or higher Syndecan-I level and variable levels of ANXA7 expressions.

We analyzed the expression of p53 and calculated the overall survival in association with ANXA7 expression. We found a subgroup of patients with poor overall and tumor-specific survival with lower p53 and higher ANXA7 expression levels (p<0.005, total cases, n = 417, **Fig 4**). The results are also consistent with earlier findings demonstrating a low prevalence of positive p53 expression [14]. The 10 year survival rate is ~ 82% at the ANXA7 expression level 1 (n = 66) and 73% at ANXA7 expression level 2 (n = 194). Whereas, a significant decrease in cumulative survival (~62%) was observed at the ANXA7 expression levels at 0 (n = 33) and 3 (n = 124). However, in this study the cumulative survivals are varied with ANXA7’s low or high expression, while p53 is inactive or very low expression **(Fig 4A**), indicating a bimodal correlation of ANXA7 with a very low p-value (p<0.005).

Our previous study indicated that the syndecan-1 overexpression predicted early recurrence and was significantly associated with high Gleason grades, Ki67 and Bcl-2 overexpression [13]. In this current study, we analyzed the correlation of ANXA7 expression with the syndecan-1 expression level **(**Overall cases for low Syndecan-1, n = 274 and for high, n = 150; **Table 1 and Fig 4B and 4C)**. We found that a lower level of syndecan-1 expression linked with low levels of ANXA7 (level 0) resulted in significantly higher cumulative survival rate (~95% survival as compared to ~50% survival for tumors that had higher ANXA7 (levels 1–3). However, a positive expression of syndecan-1 and an increase of ANXA7 level (level 3) delineated a lower survival rate (~30% for 15-year survival rate). For optimal ANXA7 expression (level 1), cumulative survival was much higher (~80%) (p<0.05, **Fig 4C**), indicating a bimodal distribution of ANXA7 expression level in terms of overall survival rate.

### Influence of neoadjuvant hormonal treatment (NHT)

**Fig 5:**
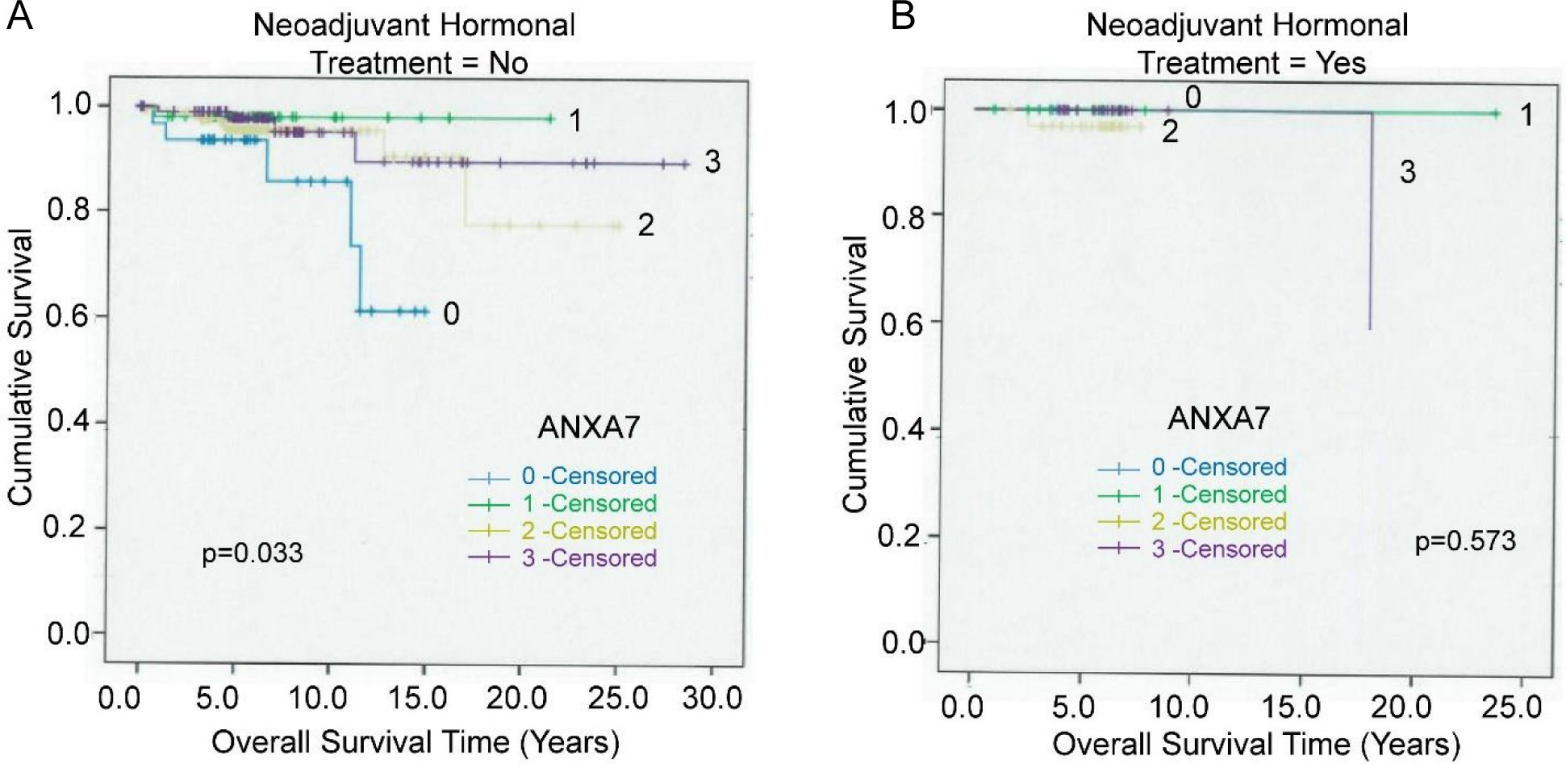
The influence of neoadjuvant hormonal treatment (NHT) with the ANXA7 based Gleason grades. The tumor-specific survival regarding NHT represented by the Kaplan-Meier curve and the higher (positive) expression of ANXA7 in relation to neoadjuvant hormonal treatment (NHT) was found. **A.** Survival curves were represented with the differential expression of ANXA7 with non-NHT cases (n = 358) and, **B**. represented the survival curves in terms of positive NHT cases (n = 77).

Next, we analyzed the correlation between ANXA7 level and survival rates in terms of neoadjuvant therapy applied to the prostate cancer patients. Neoadjuvant hormonal treatment (NHT) is the application of therapeutic agents before a main treatment. The total cases as well as the numbers related to various ANXA7 scores were presented in the **Table 1**. In our previous study, it was observed that the Ki67 and CD10 expression were both significantly lower in radical prostatectomy specimens after androgen ablation therapy than in prostatectomy specimens without prior NHT [13]. In contrast, Bcl-2 overexpression was significantly more in prostate cancers after NHT than in those without NHT [13]. To examine whether ANXA7 levels correlate with NHT response, we analyzed the expression of ANXA7 in terms of tumor specific survival in relation to NHT (**Fig 5A and 5B**).

We found that very low or negative ANXA7 levels (level 0) in patients not pre-exposed to NHT showed a sharp decrease in overall survival rates (~60%) (**Fig 5A**) with a very low p-value (p = 0.033). Whereas, NHT exposure was associated with significantly higher cumulative survival at all levels of ANXA7 (**Fig 5B**).

## Discussion

The ANXA7 is a tumor suppressor and a member of the calcium-dependent phospholipid binding proteins. However, it’s activity is frequently inactivated by genomic alterations at 10q21 [32]. The cancer-specific expression of ANXA7, coupled with its importance in regulating cell death, cell motility, and invasion, makes it a useful diagnostic marker of cancer and a potential target for cancer treatment [32]. Tumor suppressor function of the calcium/phospholipid-binding Annexin-A7 (ANXA7) has been shown in Anxa7-deficient mice and validated in human cancers, particularly for prostate cancer [23, 33]. In the androgen-resistant prostate cancer cells, ANXA7 and p53 showed similar cytotoxicity levels. However, in the androgen-sensitive LNCaP (a prostate cancer cell line), ANXA7 greatly exceeded the p53-induced cytotoxicity [33]. Therefore, the higher expression of ANXA7 should be beneficial for the prostate cancer patients. However, our current tissue microarray studies indicated that the very high expression (level 3) is not helpful for the survival of prostate cancer patients; indicating bimodal functions of ANXA7 for prostate cancer.

The application of tissue microarray (TMA) technology has generated a large number of candidate molecular markers and enables the evaluation of clinical value for potential markers. In this work, we have evaluated a candidate marker, Annexin A7 (ANXA7) expression, which was correlated with Ki67, Bcl-2, p53, CD-10, and syndecan-1 expression. A bimodal distribution of ANXA7 was observed in most of the cases in terms of cumulative survival. Tumors expressing either high or no ANXA7 were found to be associated with poor prognosis or prostate-specific antigen (PSA) recurrence was observed. However, ANXA7 at an optimal level of expression showed better prognosis.

During normal condition, in benign prostate glands, the expression of Bcl-2 and syndecan-1 was localized in the basal cell layer. Whereas, in contrast, the expression of CD-10 is cytoplasmic. As expected, in benign prostatic glands, expression of Bcl-2 and syndecan-1 was typically restricted to the basal cell layer. However, a diffuse cytoplasmic staining of CD-10 in secretory cells was observed in our previous study [13]. Besides, our previous study demonstrated that the nuclear staining of Ki67 was only present in individual scattered cells (<5%), whereas p53 was always negative in benign glands [13]. A high Ki67 (>10%) was found in 14.5% of 515 prostate cancer specimens and was associated with high Gleason grades. Ki-67 is a marker for cellular proliferation [34] and we observed a high Ki67 expression level with a Gleason grade 2 for ANXA7 (data not shown). It was confirmed to predict early progression, poor overall, and tumor-specific survival with a low Ki67 level as previously reported in whole tissue sections and core needle biopsies [13, 31, 35–37].

Bcl-2 is specifically considered as an important anti-apoptotic protein. However, ANXA7 expression in the Bcl-2 negative tumors with high Ki67 showed a bimodal characteristic in terms of overall survival. Thus, immuno-histochemical analysis to detect low levels of ANXA7 is crucial for indication of a lower order survival when both Ki67 and Bcl-2 are low (Fig 3).

Next, we also correlate our current protein of interest, ANXA7, with CD-10. CD-10 is a zinc-dependent membrane metallo-endopeptidase. This is a type II transmembrane glycoprotein and is an important cell surface marker for the diagnosis of human acute leukemia. The CD-10 cleaves peptides at the amino side of hydrophobic residues. As a result, it inactivates several peptide hormones’ activity including oxytocin, glucagon, neurotensin, enkephalins, and bradykinin. It also degrades the amyloid beta peptide which is the abnormal misfolding and aggregation in neural tissue leading to cause of Alzheimer's disease and other neurodegenerative diseases [38, 39]. We found that when CD10 expression is positive, ANXA7 expression at level-1 can significantly improve cumulative survival.

The tumor suppressor gene *TP53* (protein p53) has many mechanisms by which it prevents cancer. p53 plays a role in apoptosis, genomic stability, and inhibition of angiogenesis and its prognostic value is well established in primary prostate cancers [13, 29, 40, 41]. Consistently, a significant correlation of a bimodal distribution ANXA7 variation with p53 expression on tumor progression and cumulative survival was observed.

Our previous study had reported that syndecan-1 has prognostic significance in prostate cancer^14^. Syndecan-I is a transmembrane heparan sulfate proteoglycan and also a member of the syndecan proteoglycan family. The syndecans play a critical role in cell binding, cell signaling, and cytoskeletal organization. The basal syndecan-1 level is also crucial for understanding the progression of malignant transformation, tumor metastasis, and advanced or disseminated cancer stages [42–44]. Additionally, syndecan-1 was more frequently overexpressed in prostate cancer [13, 42, 45]. Our finding in this current study demonstrated that when syndecan-1 is positive, a higher expression of ANXA7 further decreases survival rate, highlighting the importance of ANXA7 as an important biomarker along with syndecan-1 for the prediction of survival rates in prostate cancer patients.

As mentioned earlier, ANXA7 is a tumor suppressor gene, and higher expression will be increased the overall survival of prostate cancer patients. However, our current TMA study indicated that the higher expression of ANXA7 also included the upregulation of tumor proliferation markers such as Bcl-2 and CD-10. Our current research is a further validation of our previous hypothesis and related findings; whereby, demonstrating the functional role of ANXA7 as a tumor suppressive gene. Our present high throughput TMA study extended our understanding of the role of ANXA7 towards prostate cancer patients’ survival and established its bimodal function as a tumor apoptotic marker (with optimal level) and tumor proliferative marker (with extremely low or high concentrations). The study also includes the mechanistic relevance of the ANXA7 bimodal role and its relationship with other tumors apoptotic or survival markers such as CD-10, Ki67, p53, Bcl-2 and Syndecan-1 which have high importance towards regulating prostate cancer progression.

In summary, we confirmed a prognostic significance of ANXA7 in prostate cancer and concluded that ANXA7 is an independent predictor of poor overall survival with a p-value of 0.01. We also identified a cross-talk between syndecan-1 and ANXA7 and found evidence for an androgen-dependent regulation of ANXA7. This study thus provides evidence for the importance of various ANXA7 expression levels in the determination of tumor progression and survival rates in prostate cancer patients.
